# Relative frequency of human papillomavirus genotypes and related sociodemographic characteristics in women referred to a general hospital in Tehran, 2014-2015: A cross-sectional study

**Published:** 2017-05

**Authors:** Mahin Jamshidi Makiani, Sara Minaeian, Soheila Amini Moghaddam, Seyed Akbar Moosavi, Zahra Moeini, Vajihe Zamani, Mahnaz Karbalaei Sabbagh, Hosein Forghani

**Affiliations:** 1 *Department of Infectious Disease, Firoozgar Hospital, Iran University of Medical Sciences, Tehran, Iran.*; 2 *Antimicrobial Resistance Research Center, Institute of Immunology and Infectious Diseases, Rasoul-e-Akram Hospital, Iran University of Medical Sciences, Tehran, Iran.*; 3 *Department of Gynecology Oncology, Firoozgar Hospital, Iran University of Medical Sciences, Tehran, Iran.*; 4 *Department of Genetic, Tehran University of Medical Sciences, Tehran, Iran.*; 5 *Department of Biology, Islamic Azad University of Damghan, Damghan, Iran.*; 6 *Department of Obstetrics and Gynecology, Firoozgar Hospital, Iran University of Medical Sciences, Tehran, Iran.*; 7 *Firoozgar Hospital, Iran University of Medical Sciences, Tehran, Iran.*; 8 *Department of Public Health, Shahid Sadoughi University of Medical Sciences, Yazd, Iran.*

**Keywords:** Human papilloma virus, Frequency, Multiplex PCR, Socioeconomic factors

## Abstract

**Background:**

Human papilloma virus (HPV) is one of the major public health problems and the main causes of cervical cancer. The prevalence HPV infection in developing countries with low financial resources is high.

**Objective:**

This study aimed to determine the relative frequency of HPV genotypes and its sociodemographic characteristics in women referred to a general hospital in Tehran, Iran from 2014-2015.

**Materials and Methods:**

This cross-sectional study was performed in 400 women with Pap smear samples, referring to to a general hospital in Tehran, Iran from 2014-2015. The detection of 28 HPV genotypes was performed by using the Multiplex PCR technique. The sociodemographic survey was conducted for each HPV positive woman.

**Results:**

HPV-positive infection was detected in 155 (38.75%) women aged 17-85 years. HPV 16 (19.1%) was the most prevalent type, followed by HPV 39 (12.5%) and HPV 18 (8.9%). The highest rate of HPV infection was observed at the age of 36 years (7.7%). The level of education and economic situation of each woman were showed most of HPV-positive women had a high school diploma (34.6%) and average economic situation (67,9%). 60.9% of these women were a housewife, and 67.3% lived in the capital .

**Conclusion:**

Determination of HPV genotype and risk factor related to HPV infection in each geographical region can lead to the production of effective vaccines against the HPV virus. It can also be useful for disease management and high sensitivity diagnosis of cervical intraepithelial neoplasia.

## Introduction

Human papilloma virus (HPV) is one of the major public health problems and the main causes of cervical cancer (1). It is the considerable challenge in developing countries with low financial resources (2). HPV is a member of the Papillomaviridae family (3) which is divided into high-risk, possibly high-risk, and Low-risk HPV groups. Up to now, the sequences of 150 HPV types have been determined (4). According to studies performed persistent HPV infections can lead to cervical cancer (5, 6). Some types of high risks HPV, such as HPV 16, 18, 31, 33, 35, 39, 45, 51, 52, 56, 58, 59, 66, 68 and 70 can lead to cervical carcinoma (7, 8). 

The worldwide prevalence of HPV has been varied from 2-44% (9). In a meta-analysis, which was accomplished by Bruni *et al* the global prevalence of HPV was 11.7%. The HPV prevalence was 24% in Sub-Saharan Africa, 21.4% in Eastern Europe, 16.1% in Latin America (10). According to the research that has been accomplished in Iran in 2012; the prevalence of HPV infection among women was 7% (11). Among the different types of HPV that have been observed in Iran, the prevalence of HPV 16 and HPV 18 was 7.3% and 2.8%, respectively (12). The socioeconomic situation, the level of education, living in deprived areas and regional customs and habits have a significant effect on HPV infection rate (13, 14).Smoking, alcohol consumption, and women’s sex history are some of the important risk factors of acquisition of HPV infection (15).

It has been proven HPV detection and organized screening program can be essential strategies against cervical cancer. Thus providing accurate and rapid methods for detection and identification of HPV would be very useful and helpful (16). Multiplex PCR, which is easy to use with high sensitivity and specificity, plays an important role in the detection of many viruses such as HPV (4, 17). 

By identifying risk factors contributing to HPV infection according to the customs and habits of each specific area, effective prevention methods and rapid detection of HPV infection can be achieved. It could help us to reduce the prevalence of HPV in the society significantly. Most studies within the field of HPV prevalence which has been performed in Iran have only focused on determination of the HPV types. However, in this study, we decided to survey socioeconomic and demographic characteristic’s data in addition to the relative frequency of HPV.

The main aim of this investigation was to detect the frequency of HPVs genotypes and study of risk factors associated with HPV infection in the part of the Tehran city. 

## Materials and methods

**Sample collection and DNA extraction**

In this cross-sectional study, a total of 400 women referred to the Women’s Clinic of a general hospital in Tehran, Iran during the period of one year from September 2014 to September 2015 were recruited. Our inclusion criteria were all women who referred to Women’s Clinic, needed gynecological examination and agreed to have examination. Women who were in menstrual period, pregnancy, had a history of HPV vaccination, or had a contraindication of gynecological examination were excluded. 

All participants were interviewed for their level of education, sociodemographic characteristics, age, the age of menarche, marriage or menopause, pregnancy contraception methods, underlying disease, history of sexually transmitted diseases, history of smoking, and sexual or reproductive history. Then they were undergoing a gynecologic examination and a Pap smear test by vaginal cytology brush through the liquid-based thin perb pap method. HPV DNA was extracted using the EZ DNA Methylation™ Extraction Kit (TBG, USA) according to the manufacturer’s instruction and then multiplex polymerase-chain-reaction (PCR) tests for HPV DNA were performed.

**Multiplex PCR test**

HPV genotyping was performed using the HPV-HCR Genotype-Eph kit (AmpliSens(R), Russia).The kit is based on simultaneous amplifying in one tube (multiplex-PCR) of four types of HPV DNA and allows the user to detect infections and co-infections of high-risk HPV genotypes. DNA from each HPV genotype was used as a positive control and distilled water in place of template DNA run at the same conditions as a negative control. 

Each PCR was performed in a DNA thermal cycler (Sensquest, Germany) with the following condition: The amplification was carried out with initial enzyme activation at 95^o^C for 15 min, followed by 42 cycles, including 1 sec denaturation at 95^o^C, 3 sec annealing at 63^o^C and 40 sec chain elongation at 72^o^C and a final elongation at 72^o^C for 1 min and cooling at 4^o^C. PCR products were visualized on 1.5% agarose gel by ethidium bromide staining (18, 19). 

A participant was considered HPV-positive if the test results by PCR or genotyping were positive. Samples that were negative for HPV DNA were amplified with primers for the cKi-ras gene to ensure the integrity of the samples. Samples in which neither HPV DNA nor the cKi-ras gene was amplified were considered inadequate for analysis and were excluded.

**Ethical consideration**

Informed consent was obtained from all women enrolled, and the study was approved by the Ethics Committee of the Iran University of Medical Sciences (Ref. number: IR.IUMS.REC.1391-17028).

**Statistical analysis**

Data analysis, which was obtained from Multiplex PCR test and the questionnaires were entered into an Excel spreadsheet and then data were performed using Statistical Package for the Social Sciences, version 18.0, SPSS Inc, Chicago, Illinois, USA (SPSS) software. All quantitative variables were evaluated by the mean±SEM and type-specific prevalence of HPV were summarized using frequency distributions.

## Results

**Demographic and Sociodemographic Characteristics**

The mean±SD age of participants in this study was 42.58±12.21 yr old (ranging from 17-89 yr old). Among 400 women included in this analysis, overall HPV prevalence was 38.75% [positive HPV (n=155) and negative HPV (n=245)]. The mean age of HPV-positive women was 40.47 yr old (ranging from 20-80 yr old). Also, the mean age of menarche and marriage among the HPV-infected women were 13.28±1.57 and 19.38±4.96 yr, respectively and 70.4% of them had not experienced menopause until this study was underway. The mean age of menopause among HPV-positive women was 47.29±6.93 yr. 

22.4% of HPV-positive women had never been used any contraception method (Table I). 87.2% of them did not have a history of sexually transmitted diseases (STDs), 93.6% did not have any malignancy history, and only 31.4% of them had the underlying disease. Study of the questionnaires was indicated that 0.6% were not married and were not a virgin, 87.2% married once, 7.1% had a second marriage, and 5.8% did not answer this question. Among them, 80.8% were a mother and the mean age of the first pregnancy was 20.94±4.801. 

**Figure 1:**
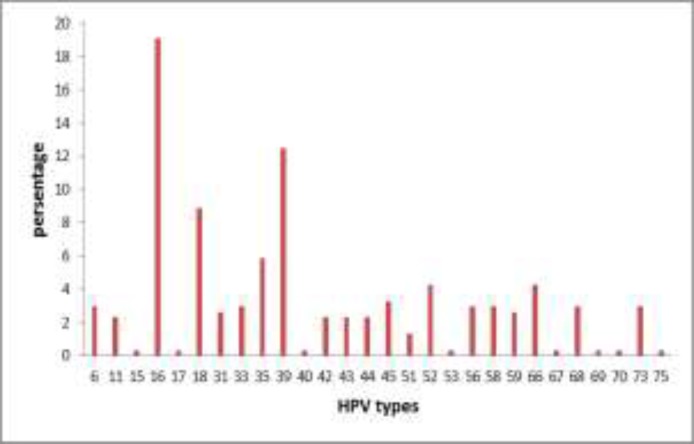
The prevalence of different HPV types among HPV DNA- positive samples

Socioeconomic analysis of HPV-positive women was performed. This means that level of education and economic situation of each were evaluated. Most of HPV-positive women had a high school diploma (34.6%) and average economic situation (67.9%). 60.9% of these women were a housewife, and 67.3% lived in the capital. A survey of the HPV-positive women showed that 96.8% of them were not smokers and 93.6% were not the hookah smokers (Table II). Twenty-eight different types of HPV were detected. The highest prevalence of HPV type was HPV16 (19.1%) and as follows HPV39 (12.5%) and HPV18 (8.9%). The lower types of HPV were HPVs 15, 17, 40, 53, 67, 69, 70, and 75 had the lowest prevalence (Figure 1).

**Table I:** Study of contraception methods in HPV-positive women

**Contraception methods**	**Frequency n (%)**
Oral contraceptive	10 (6.41)
Tubal ligation	15 (9.6)
Vasectomy	6 (3.8)
Condoms	26 (16.66)
Oral contraceptives and natural family planning	1 (0.6)
Oral contraceptives and vasectomy	1 (0.6)
Condom and natural family planning	3 (1.9)
No method	35 (22.43)
withdrawal method during intercourse	59 (37.82)
Total	156 (100)

**Table II:** Survey of smoking and Hookah smoker in HPV-positive women

	**Frequency n(%)**
Smoking	5/156 (3.2)
Hookah smoker	10/156 (6.4)

## Discussion

It has been proven persistent HPV infection can lead to cervical cancer and intraepithelial neoplasia (16, 20). Serological methods and culture are not the reliable and sensitive methods for detection of HPV, thus uses the accurate and rapid test are taken under consideration (21, 22). 

According to our results, 155/400 (38.75%) studied women were positive and infected by one or more types of HPV. Also, the most prevalent types of HPV were HPV16 (20.2%). 22 types of HPV were observed, including HPV 15, 16, 17, 18, 31, 33, 35, 39, 40, 45, 51, 52, 53, 56, 58, 59, 66, 67, 68, 70, 73 and 75.The prevalence of HPV types was changeable in different geographic regions, but HPV 16 were reported as the most prevalent and cancerous type among the other types of Human papillomavirus (23). According to studies performed in Iran, the prevalence of HPV 16 was higher than other types of HPV (24-26). In the study which was performed in Iran, the total prevalence of HPV in Iran among 7655 women was 9.4% and the prevalence of HPV 16 and 18 were respectively 2.03% and 1.7% (27).

 The prevalence of HPV 16 was 76% in 2 hospitals in Tehran, 7.3% in the other hospital in Tehran, and 15.21% in Isfahan (25, 26, 28). Clifford *et al* reported that HPV 18, 45, 31, and 33 were respectively the most common types of HPV after HPV 16 (29). In Asia, HPV 52 and 58 respectively, were more prevalent after HPV 16 and 18 (30). Our survey showed that after HPV 16, HPV 39 (16.2%) and HPV 18 (12.6%) were respectively the most prevalent type of HPV. However, Salehi-Vaziri and colleagues reported HPV 35 (9.1%) was the most common types of HPV after HPV 16 (32.8%) in Iran (24). As mentioned before, differences among the types of HPV can be due to various geographical distributions of this virus(9).

In our study, the highest rate of HPV prevalence was observed at the age 36 yr (7.7%) followed by age 46 yr (5.8%). In the study was performed by Dunne *et al* the prevalence of HPV infection was increased from aged 14-24 yr and in the older ages, the rate of HPV infection was decreased gradually. The highest rate of HPV infection prevalence was observed at the age under 20 yr. The prevalence of HPV was 19.6% in women who were in the fifties, 25.2% in forties, and 27.5% in the thirties (31). It could be due to differences in the number and type of study population. Studies in different countries indicated that the rate of HPV infection in the young women was high. It may be due to high sexual activity of them during this period of life. But the rate of HPV infection in the fourth or fifth decades of life was reduced gradually (7, 32, 33). 

However, the risk of HPV infection in women who were sexually active existed in all ages (34). Kim *et al* have reported that age was one of the significant risk factors for acquisition high-risk HPV infection and high-risk HPV infection was decreased with advancing women age (35). The results from this study indicated that in all age categories, the most infectious type was HPV 16 (19.1%), and the highest rate of infection was observed at the age of 38 years. Generally, in Iran, the mean age of menarche is reported 12.81 yr (36). 

There is much controversy over the menarche age as an independent risk factor in HPV infection (37, 38).In our research, the menarche age of most HPV-positive women was 13 years old and HPV 16 was the most prevalent type among them. The highest frequency of age in menopausal women was observed in the age 50 yr. Most of them were infected with HPV 16. The finding of the current study was consistent with Smith *et al* study who showed HPV 16 was the most prevalent type among menopausal women (39). It had shown that long duration use of oral contraception could amplify the risk of HPV infection while using a condom could reduce it (40). In the current study, HPV16 was the most common type in women who used IUD and natural withdrawal method during intercourse. In the women who had the history of the sexually transmitted disease and the history of malignancy HPV16 was the most prevalent type. HPV35 and HPV38 were detected as dominant types in women who had the underlying disease, but HPV16 was further observed in those who didn’t have. 

In Iran, the rate of cigarette smokers among women varied from 0.04-10.5%, and in comparison to the rates reported by WHO, it was the lowest rate (41). The results of this study indicated that among women who smoked and used Hookah, HPV 16 was further observed compared to other types of HPV. In study was performed in the northeast region of India HPV16 was the more prevalent type among head and neck cancer patients who had tobacco chewing habit (42). Studies have shown HPV 16 infection and use of cigarette were two significant factor, which can enhance the development of cervical cancer (43, 44). It should be noted that in Iran, smoking is not common among women.

According to the finding of Giorgi Rossi *et al* lower socioeconomic women had a little knowledge about HPV infection and cervical cancer (45). Similarly, in another study it was mentioned that lower levels of education and economic status were inversely associated with the incidence of cancers are caused by HPV infection (46). In current study women with an average socioeconomic status, had the highest rate of HPV infection and HPV 16 was observed more frequently in this group. It can be due to less referring to women who were in the lower socioeconomic status group to the physician. This evidence can also be observed in the lower literacy level of HPV-positive women. An investigation by Catarino *et al* revealed that being a housewife could be a risk factor to acquire HPV infection (47). Also, we found that 60.9% of HPV-positive women were housewives, with a high prevalence of HPV16. After that, HPV 18 and HPV 39 had the highest prevalence of HPV infection among the other type of HPV.

## Conclusion

Our study provides that HPV16 was the most prevalent type (19.1%) in HPV positive women. In all risk factors investigated in this study HPV16 was the most prevalent type. HPV genotyping and detection of risk factors associated with HPV acquisition in each region can lead to develop an effective screening program and increase the level of awareness of community members. It can also be useful for disease management and high sensitivity diagnosis of cervical intraepithelial neoplasia. Further studies should be done to investigate the HPV genotyping of the other region of Iran or the other part of the world.
